# Characterization of Composite Nano-Bioscaffolds Based on Collagen and Supercritical Fluids-Assisted Decellularized Fibrous Extracellular Matrix

**DOI:** 10.3390/polym13244326

**Published:** 2021-12-10

**Authors:** Ching-Cheng Huang, Ying-Ju Chen, Hsia-Wei Liu

**Affiliations:** 1 Department of Biomedical Engineering, Ming-Chuan University, Taoyuan City 32033, Taiwan; junas.tw@yahoo.com.tw (C.-C.H.); chyiju.tw@gmail.com (Y.-J.C.); 2 Department Life Science, Fu Jen Catholic University, New Taipei City 242062, Taiwan; 3 Graduate Institute of Applied Science and Engineering, Fu Jen Catholic University, New Taipei City 242062, Taiwan; 4 PARSD Biomedical Material Research Center, Taichung City 40749, Taiwan

**Keywords:** dermis, supercritical fluid, decellularization, nano-bioscaffold, morphology

## Abstract

Nano-bioscaffolds obtained from decellularized tissues have been employed in several medical applications. Nano-bioscaffolds could provide structural support for cell attachment and a suitable environment with sufficient porosity for cell growth and proliferation. In this study, a new combined method constitutes a decellularization protocol to remove the tissue and cellular molecules from porcine dermis for preparation of nano-bioscaffolds with fibrous extracellular matrix via pre- and post-treatment of supercritical fluids. The supercritical fluids-assisted nano-bioscaffolds were characterized by peptide identification, infrared spectrum of absorption, morphology, histological observations, DNA quantification, and hemocompatibility. Further, the resulting nano-bioscaffolds could be employed to obtain new cross-linked composite nano-bioscaffold containing collagen and acellular matrix.

## 1. Introduction

Nano-bioscaffolds containing extracellular matrix are employed for medical purposes of tissue regeneration. Numerous biomaterials for nano-bioscaffolds have been prepared and employed in various clinical applications [1,2,3,4,5,6,7,8,9,10,11,12,13,14,15,16,17,18,19,20,21,22,23,24,25,26,27]. With advantages of non-toxicity, biocompatibility and degradability, natural materials were particularly considered [3,4,5,6,7,8,9,10]. Collagen scaffolds may be entirely derived from natural sources which would provide potential advantages of specific cell interactions, hydrophilicity and biocompatibility [7,8,9,10]. However, some physical property of collagen scaffolds such as thermal property, mechanical property, and microstructure limits their applications [11]. To overcome the shortcoming, collagen scaffolds were cross-linked by chemical or physical methods or modified with natural/synthetic polymers or inorganic materials [11]. Additionally, the cross-linked natural collagen fibers would provide effective starting materials for the preparation of collagen-based bio-scaffolds which could retain their biomechanical properties [7,8,9,10,11]. The collagen-based bio-scaffold would play an essential role to provide signaling for cell attachments, cell migrations, and cell proliferations [11,12,13,14,15,16,17,18]. Decellularization of original tissues were employed in the preparation of extracellular matrix as a collagen-based bio-scaffold with biochemical constituents which could provide a collagen-based material to enhance physical property of scaffolds. Usually, decellularization methods could be chemical, physical, enzymatic methods or combined procedure of which was employed to disrupt the cells and maintain extracellular matrix structure [11,12,13,14,15,16,17,18]. Recently, supercritical carbon dioxide had been employed on the purpose of removing the cells, waste, and bioburden from tissues which could provide an alternative decellularization methods [16,17,18,19]. This study tried to establish the prospective potentials and benefits of applying physical methods such as treatments of supercritical fluids for decellularization protocols combing with the chemical method to reduce harm in essential substances of extracellular matrix as a bio-scaffold.

In this study, a new method combining pre-/post-treatments of supercritical fluids of carbon dioxide (ScCO_2_) with chemical treatments such as alkaline and enzyme for tissue decellularization was discussed and the effectiveness of the method was reported. The order and timing of the combined method constitute a decellularization protocol to obtain the desired decellularized nano-bioscaffolds with a fibrous microstructure and enhance the quality of the final acellular tissue. Residual cells would be assayed through DAPI staining test [20]. The resulting supercritical fluids-assisted nano-bioscaffold would be characterized by peptide identification, infrared spectrum of absorption, morphology, histological observations, DNA quantification, and hemocompatibility, and scanning electron microscopy (SEM). Furthermore, a new cross-linked composite nano-bioscaffolds was designed and prepared from collagen and the resulting supercritical fluids-assisted decellularized fibrous extracellular matrix. The corresponding composite nano-bioscaffolds would be characterized by Fourier transform infrared spectroscopy (FTIR), thermo-gravimetric analysis (TGA), and SEM. The resulting supercritical fluids-assisted nano-bioscaffold and its corresponding composite nano-bioscaffolds would be a potential biomedical material for medical and clinical applications.

## 2. Materials and Methods

### 2.1. Materials

L929 cells were provided by the National Infrastructure of Cell Line Resource (NSTI, Peking, China). Streptomycin and penicillin were purchased from CAISSON (Washington, DC, USA). Trypsin (sequence pure) was purchased from Promega Corporation (Madison, WI, USA). Collagen I and Collagen III standards were purchased from YO Proteins AB (Stockholm, Sweden). Fetal bovine serum (FBS), penicillin-streptomycin(100×) mixture, and 0.25% trypsin-EDTA, and Roswell Park Memorial Institute (RPMI-1640) were purchased from GIBCO (Thermo Fisher Scientific, Inc., Waltham, MA, USA). Bovine Achilles tendon type I collagen was provided from Hebei Collagen Biotechnology Co., Ltd. (Handan, China). DAPI Staining Solution, Cell Counting Kit-8 (CCK-8), Cell Cycle and Apoptosis Analysis Kit were purchased from Beyotime Biotechnology (Shanghai, China).

### 2.2. Pre- and Post-Treatments with Supercritical Carbon Dioxide (ScCO_2_)

Supercritical carbon dioxide (ScCO_2_) was used for preparation of new designed bio-scaffolds in this study. The ScCO_2_ was employed as a pre-treatment for removing most fatty acids and tissues and a post-treatment for removing residue reagents and tissues.

### 2.3. Preparation of a Collagen Scaffold by Using the New Decellularization Procedure Combining with Pre- and Post-Treatments of Supercritical Crbon Dixide (ScCO_2_)

Selection of ISO13485 quality certification, the steadily thickness of about 5 mm of dermal tissue could be obtained from porcine skin by using a designed tissue-cutting machine (Taiwan PARSD Pharm. Tech. Consulting Ltd. Co., Taichung, Taiwan). The resulting dermal tissue was pretreated by using ScCO_2_, soaked in 2 (*wt*/*v*)% NaOH_(aq)_ for 2 h with magnet mixer, and followed by 3 (*wt*/*v*)% Tritonx-100 at 25 °C for 2 h. The resulting samples were washed with double distilled water under ultrasonic wave and post-treated with ScCO_2_ to remove residual fat and organic matter. The resulting sample was frozen for 6 h and then lyophilized (EYELA, FD-5N) overnight with the use of a freeze dryer at 0.1–0.2 torr at a freeze-drying temperature of −45 °C. A designed bio-scaffold with decellularized fibrous extracellular matrix could be obtained.

### 2.4. Morphology Observations

The morphology was examined using a scanning electron microscopy (SEM, JSM6700F, JEOL Ltd., Tokyo, Japan) at 5 kV. The sample were coated with Au and scanned with SEM at a magnification between 250 and 1000.

### 2.5. Histological Examination and DNA Quantification

Before starting with the staining procedure, the sections were dewaxed and incubated with acetic acid and an Alcian blue solution. For Hematoxylin and Eosin (H&E) staining, the sections were incubated for 10 min with Harris haematoxylin solution. Five sections for each cornea were stained with eosin and Alcian blue and then observed under a Zeiss Axiovert 135 inverted microscope. Fluorescence-based quantification methods used dyes that bind to DNA, resulting in a conformational shift that produces fluorescence upon excitation. The fluorescent signal is proportional to the amount of nucleic acid present. The samples were analyzed by PicoGreen DNA fluorescence staining. Fluorescence was measured by using a fluorescence spectrophotometer F-280.

### 2.6. Hemolysis Assay

Fresh blood was drawn from healthy rabbit by employing venipuncture into tubes containing potassium oxalate anticoagulant and followed by dilution with 0.9% saline solution. The resulting nano-bioscaffold was placed into a tube containing 0.9% saline solution and then incubated at 37 °C for 30 min. Diluted blood was added into the tube and further incubated at 37 °C for 60 min. After centrifugation for 5 min at 3000 rpm, the absorbance of the supernatant at 540 nm was collected.

### 2.7. Preparation of Cross-Linked Composite Bioscaffolds Derived from Collagen and Decellularized Fibrous Extracellular Matrix Microparticles

The bovine Achilles tendon type I collagen was dispersed at a concentration of 2% (*wt*/*v*) in an acetic acid solution of 0.05 mol/mL, and stirred with a homogenizer (IKA-Werke GmbH & Co. KG, Staufen, Germany) to get a full dispersion collagen gel. The nano-bioscaffold immersed in acetic acid solution at the same concentration with collagen gel resolution were respectively placed on the table concentrator of 25 °C and 37 °C for 24 h, allowing nano-bioscaffold to fully swell in acetic acid solution to obtain a nano-bioscaffold gel. Afterwards, the nano-bioscaffold gels were added into the collagen gel following continuous stirring to obtain the composite hydrogel with a final ratio of 9:1 (*wt*/*wt*) of collagen and nano-bioscaffold. The composite gel was then injected into a custom iron plate with the size of 5 cm × 5 cm and then lyophilized. Using this method, the composite nano-bioscaffolds were obtained. After, those scaffolds were fully cross-linked by immersion in a 0.1% (*v/v*) glutaraldehyde solution (75% ethanol aqueous solution) for 1 h, freeze-dried, and sterilized at a dose of 25 kGy. Finally, the cross-linked collagen/decellularized fibrous extracellular matrix composite nano-bioscaffolds were obtained.

### 2.8. Cell Morphology on the Nano-Bioscaffolds

The resulting nano-bioscaffolds with decellularized fibrous extracellular matrix that were cut into 1 cm × 1 cm × 0.1 cm size small cubes were placed in 24-well plates, and sterilized under the cobalt 60 radiation. L929 cells were cultured using DMEM/high glucose supplemented with 10% Fetal Bovine Serum (FBS), 100 U/mL penicillin and 100 U/mL streptomycin under 37 °C, a 5% CO_2_ environment and 95% relative humidity. The L929 cell suspension was seeded on the surface of sterile nano-bioscaffolds at a density of 105 cells/well and cultured at 37 °C in a CO_2_ incubator. As a control, L929 cells were seeded at the same density on nano-bioscaffolds. After 1 day of cell seeding, the bioscaffolds were rinsed three times with PBS and fixed with 4% PFA overnight at 4 °C. The samples were rinsed with PBS thoroughly and dehydrated using a graded series of ethanol (30%, 50%, 70%, 80%, 95% and 100%) for 15 min each at room temperature. The bioscaffolds with L929 cells were dried at CO_2_ critical point and sputter-coated with platinum before viewing under the scanning electron microscopy (SEM)(JSM6700F, JEOL Ltd., Tokyo, Japan).

### 2.9. Fourier-Transform Infrared Spectra of the Bio-Scaffolds

Chemical transition of the new bio-scaffold was analyzed by Fourier-transform infrared spectroscopy (FTIR) (Nicolet iS50, Thermo Fisher, MI, USA). Transmittance values were recorded in the spectral region from 500 cm^−1^ to 4000 cm^−1^.

### 2.10. Thermo-Gravimetric Analysis (TGA)

The thermal degradation behavior of composite scaffold was recognized as the temperature at the maximum peak. Determination of the samples was carried out by TGA. TGA analysis is a technique for measuring the relationship between the mass of a substance and the temperature under a temperature control program. TGA was carried out from room temperature to 550 °C under nitrogen atmosphere. Samples of approximately 3–5 mg were placed in an alumina pot at a heating rate of 20 °C/min.

## 3. Results

### 3.1. Morphology Observations

**Figure 1:**
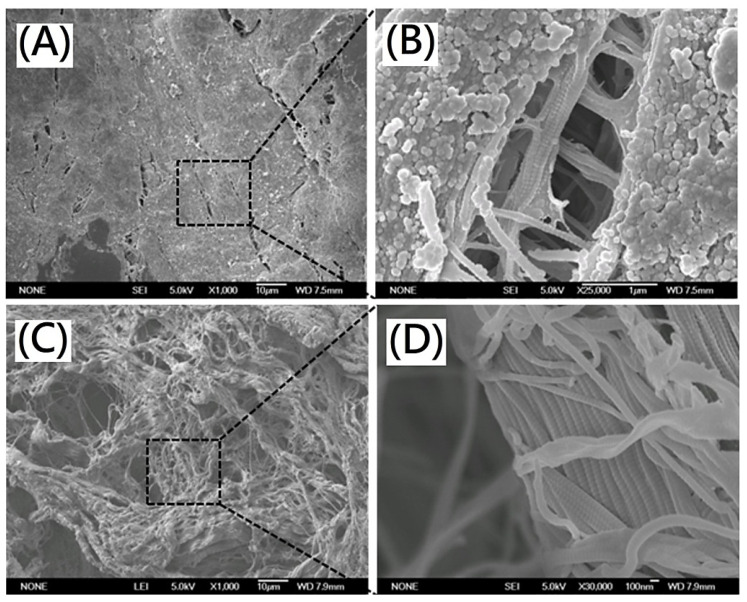
SEM images of (**A**) original porcine dermis(magnification 1000×) (**B**) original porcine dermis(magnification 25,000×) (**C**) decellularized porcine dermis after decellularization (magnification 1000×) (**D**) decellularized porcine dermis after decellularization (magnification 30,000×).

For original porcine dermis, the lipid molecules and tissues could be observed in the scanning electron microscopy (SEM) morphology (Figure 1A,B). From Figure 1C,D, the specific triple helix of the collagen as well as the fibrous microstructure of the scaffolds was observed in the SEM morphology. The quite different morphology of the resulting scaffold was observed from original porcine dermis. The lipid molecules and tissues were removed effectively. There was no apparent disruption of the overall fibrous collagen microstructure and acellular dermal matrix was maintained. The decellularized fibrous extracellular matrix containing collagen fibrils with diameters in a range of 100–200 nm was observed.

### 3.2. Histological Observations

**Figure 2:**
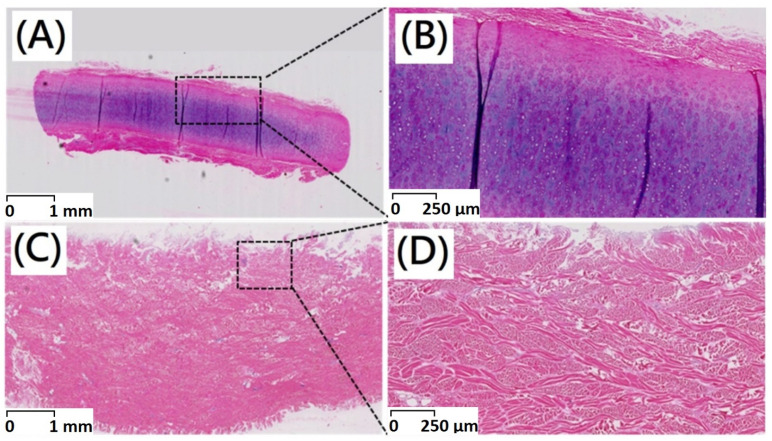
Haematoxylin–eosin staining of (**A**) original porcine dermal (scale bar indicates 1 mm) (**B**) original porcine dermal (scale bar indicates 250 μm) (**C**) porcine dermal segments after decellularization (scale bar indicates 1 mm) (**D**) porcine dermal segments after decellularization (scale bar indicates 250 μm).

**Figure 3:**
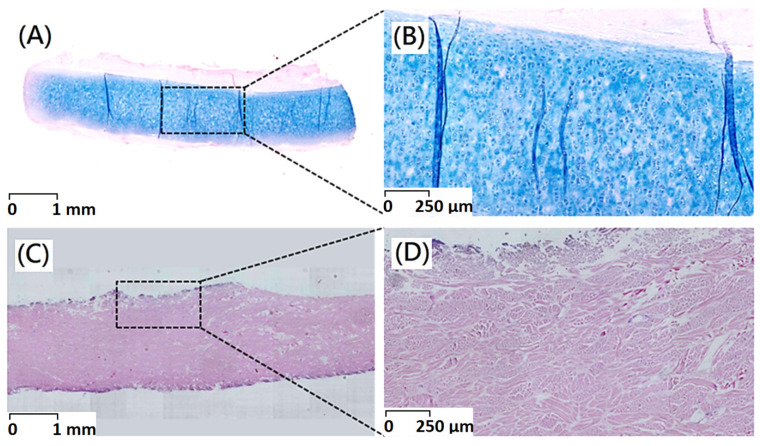
Alcian blue staining of (**A**) original porcine dermal (scale bar indicates 1 mm) (**B**) original porcine dermal (scale bar indicates 250 μm) (**C**) porcine dermal segments after decellularization (scale bar indicates 1 mm) (**D**) porcine dermal segments after decellularization (scale bar indicates 250 μm).

Histological observations could be employed to determine in vivo biocompatibility. After decellularization procedure, the majority of immunogenic porcine dermal cells were removed as shown in the results of H&E staining. An intact extracellular matrix with gross preservation of fibrous microstructure could be confirmed by Alcian blue staining. There was no apparent disruption of the intact tissue histoarchitecture and acellular dermal matrix with fibrous microstructures has been maintained (Figure 2 and Figure 3).

Hematoxylin and Eosin (H&E) staining was widely employed staining technique in histopathology. In this study, the nano-bioscaffolds of acellular dermal matrix were analyzed by histological staining. In a native porcine dermis, chondrocytes were round and embedded within lacunae as shown in Figure 2A. After decellularization procedure, no cells or cell fragments would be present in the acellular dermal matrix with fibrous microstructures (Figure 2B).

**Figure 4:**
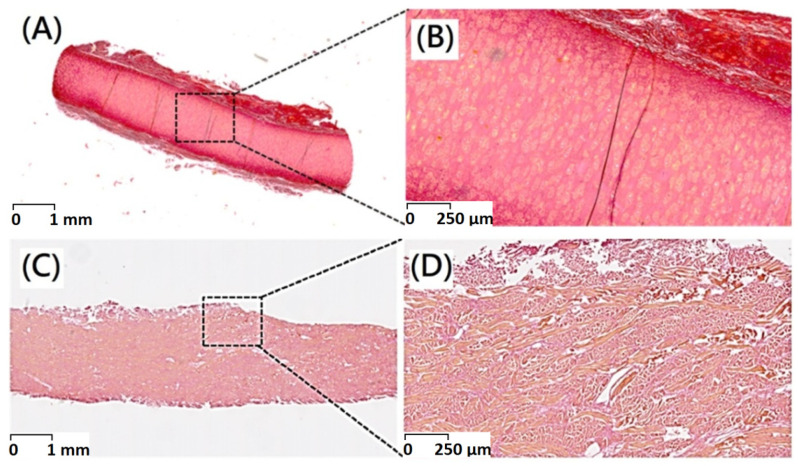
Picro-Sirius red stain of (**A**) original porcine dermal (scale bar indicates 1 mm), (**B**) original porcine dermal (scale bar indicates 250 μm), (**C**) porcine dermal segments after decellularization (scale bar indicates 1 mm), and (**D**) porcine dermal segments after decellularization (scale bar indicates 250 μm).

Picro-Sirius Red staining of native and decellularized dermal tissue demonstrating preservation of collagenous structure of dermal tissue through decellularization procedure (Figure 4).

### 3.3. Histochemical Stain

The Alcian blue staining presented with a well-organized extracellular matrix within the unaffected porcine dermis (Figure 3A,B). Alcian blue was used to visualize glycosaminoglycan (GAG), molecules that participate in biochemical signaling, play structural roles within the extracellular matrix, and support hydration of extracellular matrix. Histological examination of native porcine dermis showed positive sulphated glycosaminoglycan (sGAG) staining throughout the parenchyma and more concentrated staining within islets. From Alcian blue staining results, blue color could not be observed after decellularization procedure which indicate that GAG was removed (Figure 3C,D). A significant decrease in GAG contents would be observed after decellularization procedure.

**Figure 5:**
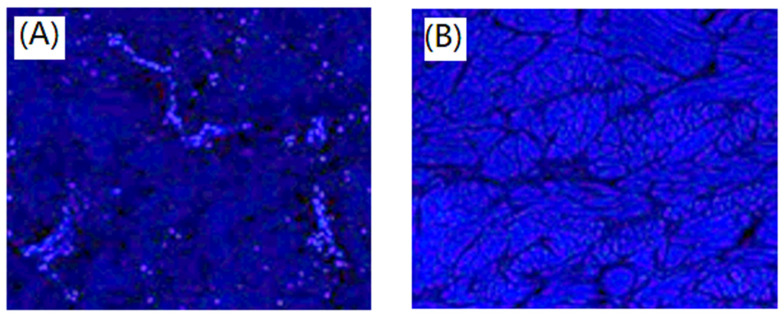
DAPI staining of unaffected porcine dermal (**A**) and porcine dermal segments after decellularization (**B**). Magnification 100×.

Residual cells were assayed through DAPI staining, the 4,6-diamidino-2-phenylindole (DAPI) staining showed nuclei labelled cells in the unaffected porcine dermal. The dermal cells were observed in Figure 5A. The cellular nuclei were visible indicating existence of host cells. After decellularization, DAPI staining showed nearly complete removal of dermal cells as shown in Figure 5B. Cell nuclei in fibroblast cell sheet exhibited normal round shape and bright blue DAPI staining. In this study, a new protocol containing pre- and post- treatments was optimized to produce an acellular porcine dermal scaffold and investigated its mechanical integrity and biocompatibility. Histological analysis of the acellular matrix with fibrous microstructures showed that the dermal cells had been removed substantially, collagen fibers were kept arranged in nano-bioscaffolds were still intact. Additionally, it could be observed in the SEM morphology results as shown in Figure 1. SEM morphology, Haematoxylin and eosin, Alcian blue staining, and Picro-Sirius red staining illustrated relative maintenance of matrix, especially collagen and architecture. Similar behaviors were observed in porcine pericardial tissue by Choe et al. [20].

### 3.4. DNA Quantification

**Figure 6:**
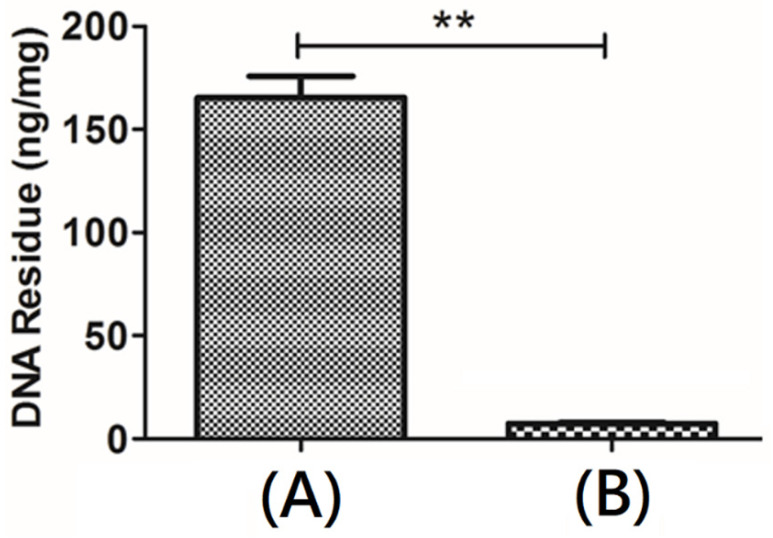
Quantitative DNA detection of (**A**) original porcine dermal and (**B**) decellularized bio-scaffold (Data are mean ± SD, n = 3; ** *p* < 0.01).

Quantitative DNA detection would reflect the amount of nucleic acid residue in a scaffold material, and it could be used as evaluation information for the biocompatibility of the scaffold material. According to the instructions of PicoGreen fluorescent dyestuff, the fluorescence intensity and DNA concentration could be plotted as a standard curve (Figure 6). After pretreatment with ScCO_2_, the lipid molecules and barriers would be extracted from the microstructure of native porcine dermis and the DNA double strands were exposed. The amount of DNA in the ECM scaffolds after decellularization was lower than 1%. The cytoplasmic protein β-actin was absent from the treated sample. Compared with native porcine dermis, the DNA content changed significantly. An approximately over 99% reduction in the DNA content of the resulting decellularized bio-scaffold was observed, which could be due to the hydrolysis of histone associated with DNA. This result indicated that the procedure containing pre-and post-treatments with ScCO_2_ could remove cells effectively. The free DNA molecules would be shed from decellularized tissue during decellularization via ScCO_2_. The decellularized reagents such as NaOH and Triton would degrade connective tissue proteins into small peptides and even amino acids. The remarkably removal of DNA was observed.

### 3.5. Hemocompatibility

**Figure 7:**
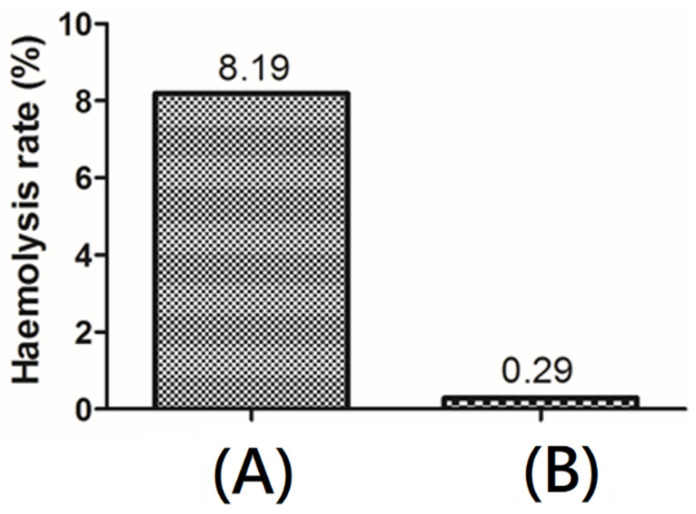
Hemocompatibility of (**A**) original porcine dermal and (**B**) decellularized bio-scaffold.

The contact of red blood cells with materials throughout their life cycle may significantly affect their inherent functions. The hemolysis percentage denotes the degree of red blood cells broken by the test sample in contact with blood. The hemocompatibility of the resulting decellularized bio-scaffold containing acellular matrix with fibrous microstructures was assessed by hemolysis. The results revealed that the hemolysis percentage was lower than 0.5% for bio-scaffold after decellularization procedure (Figure 7), demonstrating good antihemolysis characteristic of the resulting decellularized bio-scaffold.

### 3.6. Preparation of Deigned Cross-Linked Decellularized Extracellular Matrix/Collagen Composite Nano-Bioscaffolds

**Figure 8:**
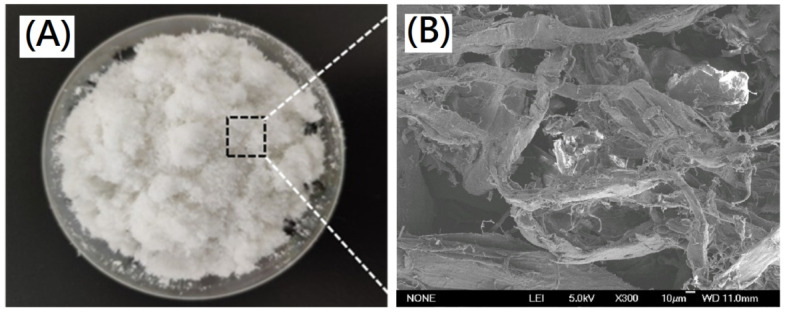
(**A**) Photograph of the resulting decellularized nano-bioscaffold microparticles and (**B**) SEM photograph of the resulting decellularized nano-bioscaffold microparticles(scale bar indicates 10 µm).

**Figure 9:**
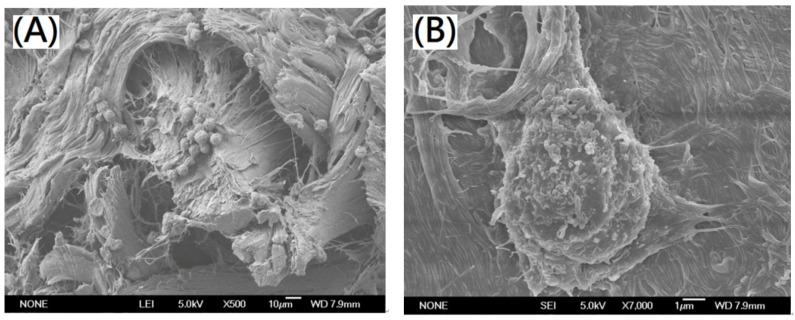
SEM photographs of L929 cells grew on the resulting decellularized nano-bioscaffold microparticle with magnification (**A**) 500× (scale bar indicates 10 µm) (**B**) 7000× (scale bar indicates 1 µm).

For preparation of designed cross-linked collagen/decellularized extracellular matrix composite nano-bioscaffolds, the decellularized nano-bioscaffold containing acellular matrix with fibrous microstructures were further powdering to nano-bioscaffold microparticles as shown in Figure 8A,B. The fibrous acellular matrix containing collagen fibrils with diameters in a range of 0.1~1.0 μm and collagen fibers with diameters in a range of 10~50 μm was observed in the nano-bioscaffold microparticle. In previous work, the pore space of nano-bioscaffold was suggested to be small enough to establish a high specific surface area and large enough to allow cells to migrate into the microstructure (20~120 μm) [21]. In this study, the pore space of the resulting nano-bioscaffold with diameters in a range of 10~250 μm could be observed which might be a good bioscaffold for cell migration [21]. Further, L929 cells were cultured on the nano-bioscaffold microparticles. The morphology of L929 cells cultured on the resulting microparticles was investigated by SEM as shown in Figure 9A,B. Significantly, most area was covered by L929 cells on the resulting decellularized nano-bioscaffold microparticles after three day. The attached cells on the microparticles after three day of culture were observed by confocal microscopy. The cells which grew upon the resulting scaffold were significantly observed.

### 3.7. Morphology and Thermal Properties of Composite Nano-Bioscaffolds from Collagen/Decellularized Bio-Scaffold Containing Acellular Matrix with Fibrous Microstructures

The microparticles and type I bovine tendon collagen were immersed, dispersed in acetic acid solution to get a composite bio-scaffold hydrogel with a ratio of 9:1 (*wt*/*wt*) of collagen and decellularized bio-scaffold containing acellular matrix with fibrous microstructures. The designed composite nano-bioscaffolds could be obtained from collagen and decellularized bioscaffolds with fibrous extracellular matrix, which was prepared by using a combination procedure of lyophilization and crosslinking reaction. For the characterization of the resulting composite nano-bioscaffolds, functional groups of amide I, amide II, amide III, amide A, and amide B could be identified corresponding to collagen in the FTIR spectra. The five characteristic signals of collagen and protein could be observed in the same range as the ones carried out in previous works [21,22,23] In this study, the resulting composite nano-bioscaffolds containing collagen showed the band of 1650 cm^−1^ (CO) corresponding to Amide I vibration, the band of 1548 cm^−1^ corresponding to amide II vibration δ(NH)/ν(CN), the band of 1237 cm^−1^ corresponding to amide III vibration δ(NH)/ν(CN), the band of 3296 cm^−1^ for amide A vibration (NH), and the band of 2922 cm^−1^ corresponding to amide B vibration (CH_2_). Those functional groups are corresponding to collagen.

**Figure 10:**
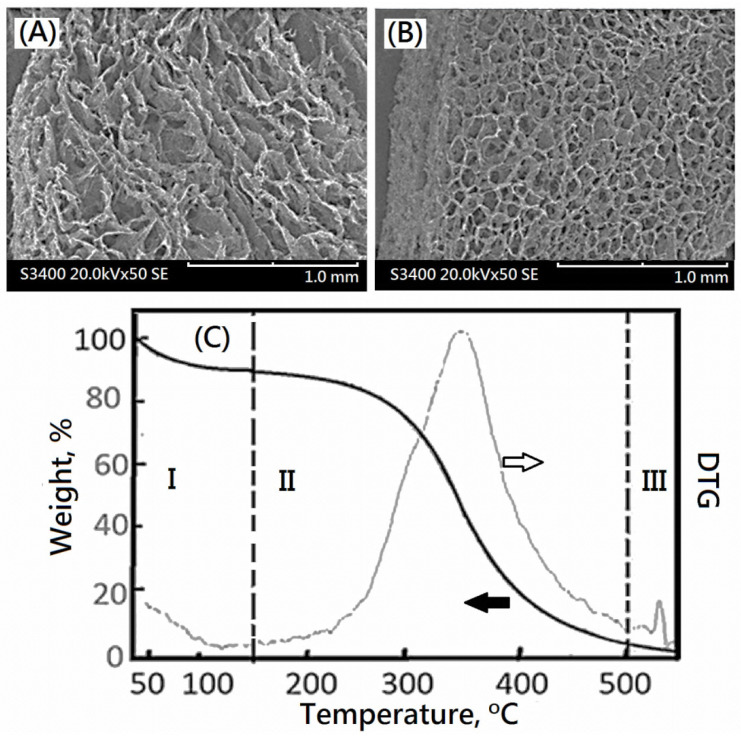
The SEM images of (**A**) collagen nano-bioscaffold and (**B**) designed composite nano-bioscaffold, and (**C**) the weight percent and DTG profiles of designed composite nano-bioscaffold.

**Figure 11:**
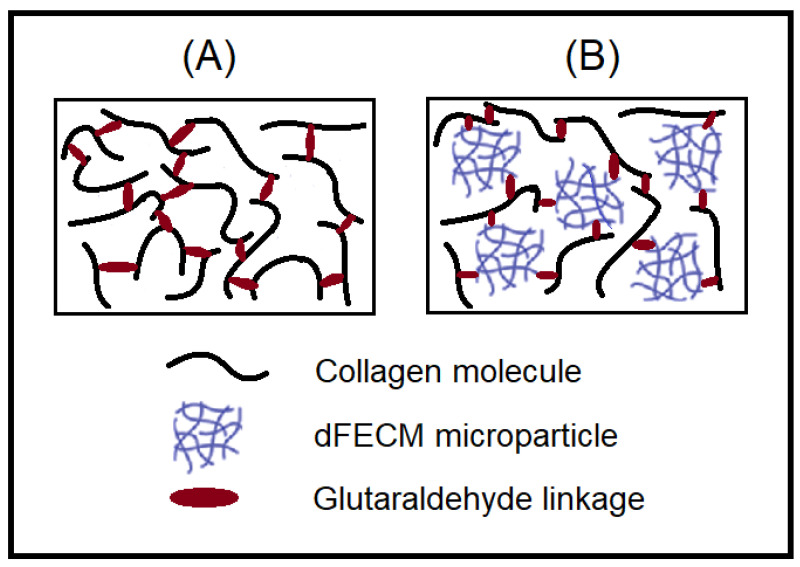
Schematic diagram for preparation of designed crosslinked composite bioscaffold. (**A**) cross-linked collagen and (**B**) cross-linked collagen and decellularized bio-scaffolds.

Furthermore, SEM morphologies of a cross-linked collagen and the corresponding composite nano-bioscaffolds of cross-linked collagen/decellularized bioscaffolds were observed in Figure 10A,B, respectively. The compacted porous microstructure was observed. The addition of glutaraldehyde would form the interconnection of the porous microstructure. The similar compacted porous microstructure of cross-linked collagen or gelatin with glutaraldehyde could also be observed in the previous work [22]. In this study, the microstructure with relative uniform and small porosity (diameter ca.0.1 mm) could be observed in the SEM morphology of the resulting cross-linked collagen/decellularized bioscaffolds. Additionally, the relative high crosslinked density was observed as shown in Figure 10B. It might be a formation of colloidal dispersion of bio-scaffold microparticle and collagen molecules before cross-linking reactions and a uniform distribution of compacted microstructure of bio-scaffold microparticle as shown in schematic diagram (Figure 11).

The TGA provided information about the behavior with the temperature presented by the resulting composite nano-bioscaffold of cross-linked collagen and decellularized bio-scaffolds containing acellular matrix as shown in Figure 10C. The main loss is presented in three different temperature ranges given by temperature region I: (<150 °C); temperature region II: (150–500 °C) and temperature region III:(>500 °C), as shown in Figure 10C. The decrease trend of the weight loss curve in region I would be resulted from the loss of the physisorbed water in the resulting composite nano-bioscaffold, which represented the 10 wt% of the collagen complex bioscaffold, which occurred at 40 °C. The following maximum pyrolysis temperature (T_d,max_), occurring in the temperature region II in the thermogram, for the resulting composite nano-bioscaffold was observed at 347 °C which is higher than the maximum pyrolysis temperature(T_d,max_) of the corresponding bio-scaffold microparticle(ca.339 °C). The weight losses of temperature region II were related to the combustion of the resulting composite nano-bioscaffold of cross-linked bio-scaffold/collagen. The DTG curve with single peak implied the uniform microstructure of cross-linked bio-scaffold/collagen was obtained after cross-linking reactions. In previous work, pure collagen exhibited decomposition with the inflection points at 320–331 °C because of the destruction of the macromolecular structure of collagen [26,27]. The resulting composite nano-bioscaffold exhibited a better thermal stability than collagen.

## 4. Conclusions

Cross-linked composite nano-bioscaffolds derived from collagen and decellularized fibrous extracellular matrix microparticles could be successfully designed and obtained by using a novel process combining pre- and post-treatments with supercritical fluids. The characteristic of cross-linked composite nano-bioscaffolds were determined for preclinic evaluation of medical applications. The SEM morphology of a cross-linked collagen and a cross-linked composite nano-bioscaffolds could be observed. The microstructure with relative uniform and small porosity (diameter ca.0.1 mm) and relative high crosslinked density could be observed in cross-linked composite nano-bioscaffolds. The T_d,max_ for the cross-linked composite nano-bioscaffolds was observed at 347 °C which is higher than the T_d,max_ of nano-bioscaffold microparticle(ca.339 °C). These losses are related to the combustion of cross-linked composite nano-bioscaffolds. The DTG curve with single peak implied the uniform microstructure was obtained after cross-linking reactions.

## Data Availability

The data presented in this study are available on request from the corresponding author.
